# Age- and Sex-Matched Normal Leukocyte Subset Ranges in the General Population Defined with the EuroFlow Lymphocyte Screening Tube (LST) for Monoclonal B-Cell Lymphocytosis (MBL) vs. Non-MBL Subjects

**DOI:** 10.3390/cancers15010058

**Published:** 2022-12-22

**Authors:** Ignacio Criado, Wendy G. Nieto, Guillermo Oliva-Ariza, Blanca Fuentes-Herrero, Cristina Teodosio, Quentin Lecrevisse, Antonio Lopez, Alfonso Romero, Julia Almeida, Alberto Orfao, The Primary Health Care Group of Salamanca for the Study of MBL

**Affiliations:** 1 Translational and Clinical Research Program, Centro de Investigación del Cáncer (IBMCC; CSIC–Universidad de Salamanca); Cytometry Service, NUCLEUS; Departamento de Medicina, Universidad de Salamanca and Institute of Biomedical Research of Salamanca (IBSAL), 37007 Salamanca, Spain; 2 Biomedical Research Networking Centre Consortium of Oncology (CIBERONC), Instituto de Salud Carlos III, 28029 Madrid, Spain; 3 Centro de Atención Primaria de Salud Miguel Armijo, Sanidad de Castilla y León (SACyL), 37007 Salamanca, Spain

**Keywords:** flow cytometry, EuroFlow, lymphocyte screening tube (LST), leukocyte subsets, reference ranges, normal cells, blood, age, sex, monoclonal B-cell lymphocytosis (MBL)

## Abstract

**Simple Summary:**

Assessment of the status of the immune system in both health and disease requires robust and reliable reference ranges for the different blood leukocyte (sub)populations that take into consideration factors that might influence their distribution, such as age, sex, ethnicity and the presence vs. absence of low-count monoclonal B-cell lymphocytosis with a chronic-lymphocytic-leukemia-like phenotype (MBL^lo^). It should be noted that despite MBL^lo^ being highly prevalent in the general population and being associated with immune impairment, MBL^lo^ individuals have not been previously excluded in the definition of normal leukocyte ranges. Here, we provide reference cell-count ranges for the major leukocyte populations identified in blood using an optimized and fully validated 8-color flow-cytometry antibody combination based on the largest (*n* = 706) cohort reported to date of Caucasian adult donors from the general population, grouped by age and sex, and highlight the altered immune profiles associated with MBL^lo^ (622 non-MBL and 84 MBL^lo^ subjects).

**Abstract:**

Reference ranges of blood-circulating leukocyte populations by, e.g., age and sex, are required for monitoring immune-cell kinetics. Most previous reports in which flow cytometry has been used to define the reference ranges for leukocyte counts included a limited number of donors and/or cell populations and/or did not consider age and sex simultaneously. Moreover, other factors not previously considered in the definition of normal ranges, such as the presence of chronic-lymphocytic-leukemia (CLL)-like low-count monoclonal B-cell lymphocytosis (MBL^lo^), might also be associated with an altered distribution of leukocytes in blood in association with an immunodeficiency and increased risk of infection and cancer. Here, we established reference cell-count ranges for the major populations of leukocytes in blood of non-MBL and MBL^lo^ adult Caucasians matched by age and sex using the EuroFlow Lymphocyte Screening Tube (LST). A total of 706 Caucasian adult donors—622 non-MBL and 84 MBL^lo^—were recruited from the general population. Among non-MBL donors, the total leukocyte, neutrophil, basophil dendritic cell and monocyte counts remained stable through adulthood, while the absolute numbers of T- and B-cell populations and plasma cells decreased with age. The number of eosinophils and NK-cell increased over time, with clear differences according to sex for certain age ranges. In MBL^lo^ subjects, few differences in the absolute cell counts by age (vs. non-MBL) were observed, and MBL^lo^ men and women showed similar trends to non-MBL subjects except for the B-cell count drop observed in >70 y-men, which was more pronounced in MBL^lo^ vs. non-MBL controls. Building robust age- and sex-matched reference ranges for the most relevant immune-cell populations in the blood of non-MBL donors is essential to appropriately identify an altered immune status in different clinical settings and highlight the altered immune-cell profiles of MBL^lo^ subjects.

## 1. Introduction

White blood cells (WBC) are key components of the immune system for which blood acts as a crossroads where recently produced cells arrive, recirculate and migrate to distinct tissues. Thus, assessment of the blood distribution of different populations of circulating leukocytes is commonly used for monitoring immune responses in both physiological and diverse pathological conditions, including aging, immunodeficiency, autoimmunity, infection and cancer [1], among others.

Currently, flow cytometry (FC) is the method of choice for the identification and further subsetting of the numerous populations of blood-circulating immune cells. However, the identification of altered immune-cell profiles requires the use of standardized antibody panels, sample preparation techniques, data acquisition and data analysis tools as well as pre-defined normal ranges [2]. Previous studies have shown that the distribution and absolute cell counts of distinct populations of leukocytes in blood fluctuate depending on age, sex and ethnicity, among other factors [3,4,5,6,7,8,9,10,11,12,13]. However, few reports have thoroughly documented in-depth the age- and sex-associated changes in the counts of blood immune-cell subsets throughout life [3,4,5,8,9,11,12,13]. However, most of these reports, including those in which FC has been used for the definition of reference ranges of various leukocyte populations, are based on a limited number of individuals [3,4,5,7,10], and they do not provide reference values according to both sex and age ranges at the same time. Additionally, they analyzed a small number of cell populations, or the FC technique applied was not fully optimized and standardized [3,4,5,10]. Since the availability of normal ranges for the different subsets of blood leukocytes in healthy subjects and their variations through life is a prerequisite for robust identification of altered disease-associated immune-cell profiles, to overcome these limitations, further studies in larger cohorts of individuals are needed, in which standardized FC techniques are used for the robust definition of reference ranges for a large number of circulating leukocyte populations according to all the above referred variables.

In turn, previous studies have shown frequencies of 3.5–17% [14,15,16,17,18,19,20,21] cases carrying monoclonal B-cell lymphocytosis (MBL) in the general adult population, in association with altered B-cell profiles and other circulating immune-cell alterations [22,23]. Despite this, the presence of MBL^lo^ has never been taken into consideration to establish normal leukocyte reference ranges for various populations of blood immune cells.

Following its first description in 2005 [24], MBL is currently defined by the WHO [25] as the presence of low numbers (<5 × 10^9^ cells/L) of clonal mature B cells in blood with a phenotype that usually resembles (in our setting in Europe and the United States) that of chronic lymphocytic leukemia/small lymphocytic lymphoma (CLL/SLL-like MBL) [25]. Based on the overall clonal B-cell count in blood, CLL/SLL-like MBL is further subdivided into low-count MBL (MBL^lo^ or clonal B-cell expansion) and high-count MBL (MBL^hi^) (now termed CLL/SLL-type MBL [25]) depending on whether clonal B cells represent <0.5 × 10^9^ cells/L or ≥0.5 × 10^9^ cells/L, respectively [25,26]. Whereas MBL^hi^ subjects usually show lymphocytosis, MBL^lo^ presents with normal leukocyte and lymphocyte counts, the latter being a highly prevalent condition in the general population, particularly among adults aged >40 y (between 3.5% and 17%, depending on the sensitivity of the FC approach used), as identified in population-based screening programs [14,15,16,21]. Because of this, a substantial number of MBL^lo^ cases (if not all) have been included in most previous reports in the calculation of normal reference ranges for total leukocytes and their subsets. However, MBL^lo^ detected in the general population has been associated with both an altered distribution of immune-cell populations in blood and a significantly higher rate of infections and cancer, including also in some reports, a shorter life expectancy vs. age- and sex-matched non-MBL subjects from the same geographical area [20,23,27]. Among other alterations, MBL^lo^ subjects have been reported to display significantly decreased absolute counts of recently-produced immature and naïve pre-germinal center (pre-GC) B cells, together with increased CD8^+^ T-cell counts [22]. However, these alterations could not be confirmed in other studies, which did not find significantly different pre-GC B-cell counts in the blood of MBL^lo^ vs. non-MBL subjects [20,23] while showing an overall increase in total T-cell (but not CD8^+^ T-cell) and NK-cell counts in blood [20]. Such discrepancies are likely related to the more limited sample size of the later studies. Therefore, further investigations of larger cohorts of healthy adults are needed to better define the distribution of these and other leukocyte populations in the blood of MBL^lo^ vs. non-MBL subjects.

Here, we define robust age- and sex-matched reference ranges for the major populations of blood leukocyte subsets in healthy Caucasian adults from the general population living in the Castilla-León region in northwest Spain, including separate reference values for adults presenting with and without MBL^lo^, in order to highlight specifically altered immune cell profiles of MBL^lo^ subjects.

## 2. Materials and Methods

### 2.1. Donors and Samples

A total of 706 adult Caucasian healthy donors—320 men and 386 women—recruited between 2007 and 2019 from the same geographical area (Salamanca province, northwest Spain) were screened for the presence of MBL clones in blood (Supplementary Table S1). Those individuals with prior diagnosis of hematological neoplasia/disease and/or ongoing infection, active allergy or immunomodulatory treatment at sampling, as well as those with MBL^hi^, CLL or another mature peripheral blood B-cell neoplasm, were excluded from the analysis.

Before entering the study, each subject gave his/her written informed consent to participate according to the guidelines of the Declaration of Helsinki after the study had been approved by the local institutional Ethics Committee (University Hospital of Salamanca/IBSAL; approval codes: CEA Res26/02/2007 and CEIC PI4705/2017).

### 2.2. Flow Cytometry Immunophenotypic Studies

Overall, 800 µL of EDTA-anticoagulated blood/donor was used to screen for MBL based on the EuroFlow Lymphocyte Screening Tube (LST) (Supplementary Table S2) [28,29,30] A first version of the tube, which did not include the anti-TCRγδ, was applied for samples collected between 2007 and 2011, while second and third (complete) versions of LST already containing anti-TCRγδ reagent in a liquid vs. dried antibody format were used for samples collected in 2012–2017 and 2018–2019, respectively (Supplementary Table S2). For sample preparation, the EuroFlow stain-lyse/fix-and-then-wash standard operating protocol (SOP) was strictly followed after two washing steps with phosphate-buffered saline (PBS) to remove plasma-soluble immunoglobulins (Ig), as described in detail in the Supplementary Materials data.

For each sample, 5 × 10^6^ leukocytes were acquired in a FACSCanto II flow cytometer—Becton Dickinson Biosciences (BD), San Jose, CA, USA—using the FACSDiva software (BD). Instrument setup and calibration, fluorescence compensation, and daily monitoring of instrument performance were all carried out following the EuroFlow SOP [28,29] available at www.euroflow.org. For data analysis, the Infinicyt software (Cytognos SL, Salamanca, Spain) was used following a standardized gating strategy, as described elsewhere [30]. Absolute cell counts/µL of blood were calculated using a dual-platform approach, as previously described [31]. MBL^lo^ clones were identified based on the presence of a CD19^+^ B-cell population co-expressing CD20^+lo^ and CD5^+^ with restricted usage of (low levels of) Ig light-chain (i.e., kappa or lambda Ig light-chain restriction) on their surface membrane whenever the absolute number of clonal B cells was of <0.5 × 10^9^ cells/L [32].

### 2.3. Statistical Analyses

All statistical analyses were performed using SPSS V23 (IBM, Armonk, NY, USA), MIDAS v2.0.5.d (Cytognos SL, Salamanca, Spain) and GraphPad Prism V8 (GraphPad Software, San Diego, CA, USA). The Kolmogorov–Smirnov test was used to assess the distribution of data for continuous variables. As all continuous variables evaluated followed a non-Gaussian distribution or the number of cases to be compared was limited (*n* ≤ 20), the Mann–Whitney U or the Kruskal–Wallis non-parametric tests for 2 or >2 independent samples, respectively, were used. Corrected (Benjamini–Hochberg procedure) *p* values ≤0.05 for multiple comparisons to test for differences across all age groups with a false discovery rate (FDR) of <10% were considered to be associated with statistical significance. Normalization of leukocyte values by age (Supplementary Methods File S1) and graphical plotting of the data were performed using MIDAS (v2.0.5.d) (Cytognos SL).

## 3. Results

### 3.1. Kinetics of the Major Blood Leukocyte Populations in Non-MBL Healthy Donors (HD) through Adulthood, According to Age and Sex

**Figure 1:**
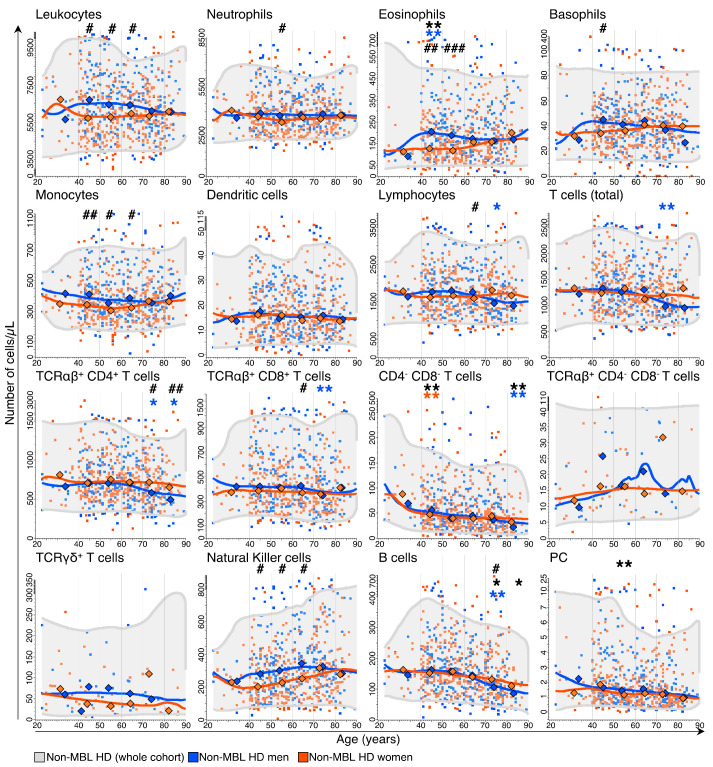
Distribution of the major populations of blood leukocytes identified with the LST antibody combination in non-MBL healthy (HD) donors grouped by age and sex. Blue and orange dots and diamonds correspond to individual data points and median values for non-MBL men and women, respectively. The horizontal grey area represents equally weighted 5th–95th percentile ranges, while blue and orange lines represent the equally weighted median values for men and women, respectively. Vertical lines delineate age ranges. Statistically significant differences in the absolute number of cells vs. the previous age group are depicted with asterisks (*p*-value: * <0.05 and ** ≤0.01, where black, blue and orange asterisks correspond to the whole cohort, men and women, respectively). Statistically significant differences between men and women (within the same age group) are indicated with hashtags (*p*-value: # <0.05, ## ≤0.01 and ### ≤0.001).

Cell counts for the major leukocyte populations evenly fluctuated throughout life in the non-MBL HD. Briefly, leukocyte, neutrophil, basophil, monocyte and dendritic cell counts remained stable in blood through adulthood, independently of age (Figure 1). Conversely, the absolute number of eosinophils, T, B and NK cells as well as plasma cells varied significantly among age groups (Supplementary Figure S1). Thus, eosinophil counts showed a consistent trend to increase with age (Supplementary Figure S1), particularly between individuals aged 40–49 y vs. the 18–39 y age group (median: 176 cells/μL vs. 104 cells/μL [*p* = 0.002]) (Figure 1 and Supplementary Table S3). In this same line, NK-cell counts progressively increased through adulthood, particularly among women, with the most striking differences found when comparing the cell counts of the youngest age groups (i.e., 18–59 y) against the 70–79 y age group (Supplementary Tables S3 and S4 and Figure S1). In turn, the CD4^+^ T-cell (Supplementary Figure S1) and CD4^−^CD8^−^ T-cell counts gradually decreased through adulthood, with such decreases being particularly significant between the ages of 18–39 y vs. 40–49 y and 70–79 vs. ≥80 y (median: 79 cells/μL vs. 51 cells/μL and 43 cells/μL vs. 27 cells/μL, respectively [*p* ≤ 0.009]) (Figure 1 and Supplementary Table S3). Similarly, B-cell numbers also decreased progressively in individuals ≥70 y (median: 140 cells/μL in donors aged 60–69 y vs. 121 cells/μL in 70–79 y age group and 102 cells/μL in ≥80 y subjects [*p* < 0.031]) (Figure 1 and Supplementary Table S3 and Supplementary Figure S1), while plasma cells showed progressively decreased numbers throughout adulthood (Supplementary Figure S1), with a more profound decrease in the fifth decade of life (median at 50–59 y of 1.2 cells/μL vs. 1.7 cells/μL for donors aged 40–49 y [*p* = 0.006], respectively) (Figure 1 and Supplementary Table S3).

Regarding gender, virtually all leukocyte subsets showed differences in their blood counts between men and women at specific age ranges. Thus, men showed significantly higher numbers of total leukocytes as well as all of the innate-cell subsets except dendritic cells, including transiently higher WBC counts in men vs. women from the age of 40 y to 70 y (Figure 1 and Supplementary Table S4). This same profile was also observed for the absolute neutrophil counts in men aged 50–59 y (median: 3696 cells/μL vs. 3330 cells/μL in 50–59 y women [*p* = 0.031]) and for both the eosinophil and basophil counts in men aged 40–49 y (median: 206 eosinophils/μL and 45 basophils/μL vs. 128 eosinophils/μL and 34 basophils/μL in 40–49 y women [*p* < 0.005]) (Figure 1 and Supplementary Table S4). Interestingly, eosinophil counts remained steadily higher in men than women until 60 years of age, similarly to monocyte and NK-cell counts, which were significantly higher among men vs. women aged 40–69 y (Figure 1 and Supplementary Table S4).

In turn, lymphocyte counts slightly decreased over time in men at the expense of both T and B cells (Supplementary Figure S1). This was particularly due to lower TCRαβ^+^CD4^+^ cells in individuals aged ≥70 y (median: 717 cells/μL vs. 578 cells/μL vs. 493 cells/μL for 60–69 y vs. 70–79 y vs. ≥80 y men, respectively, [*p* ≤ 0.029]) and of TCRαβ^+^CD8^+^ cells in the 70–79 y range vs. the previous age group (median: 344 cells/μL vs. 429 cells/μL [*p* = 0.008], respectively) (Figure 1 and Supplementary Table S4). However, when considering differences in lymphocyte counts by sex, similar total lymphocyte counts were found in men vs. women, except for individuals aged 60–69 y, in which higher lymphocyte numbers were found in men (median: 1743 cells/μL vs. 1582 cells/μL [*p* = 0.035], respectively) (Supplementary Table S4) at the expense of TCRαβ^+^CD8^+^ cells (median: 429 cells/μL in men vs. 374 cells/μL in women [*p* = 0.014]) (Supplementary Table S4). In contrast, the number of TCRαβ^+^CD4^+^ cells was significantly lower among men aged ≥70 y (median: 578 cells/μL vs. 717 cells/μL in women for individuals aged 70–79 y [*p* = 0.037], and 493 cells/μL vs. 646 cells/μL for men vs. women aged ≥80 y [*p* = 0.002], respectively) (Figure 1 and Supplementary Table S4).

Similar to T cells, the significantly decreased B-cell counts observed in non-MBL HD ≥70 y (Figure 1) was particularly lower among men aged 70–79 y compared to the previous age group (median: 104 cells/μL vs. 144 cells/μL, respectively [*p* = 0.007]) (Figure 1 and Supplementary Table S4). Of note, at this age range, B-cell counts were significantly lower in men vs. women (median: 104 cells/μL vs. 132 cells/μL [*p* = 0.005]) (Figure 1 and Supplementary Table S4), with a similar trend also for individuals aged ≥80 y (median: 83 cells/μL in men vs. 110 cells/μL in women, respectively [*p* = 0.096]) (Figure 1 and Supplementary Table S4).

### 3.2. Kinetics of the Major Blood Leukocyte Populations in MBL^lo^ Subjects through Adulthood According to Age and Sex

**Figure 2:**
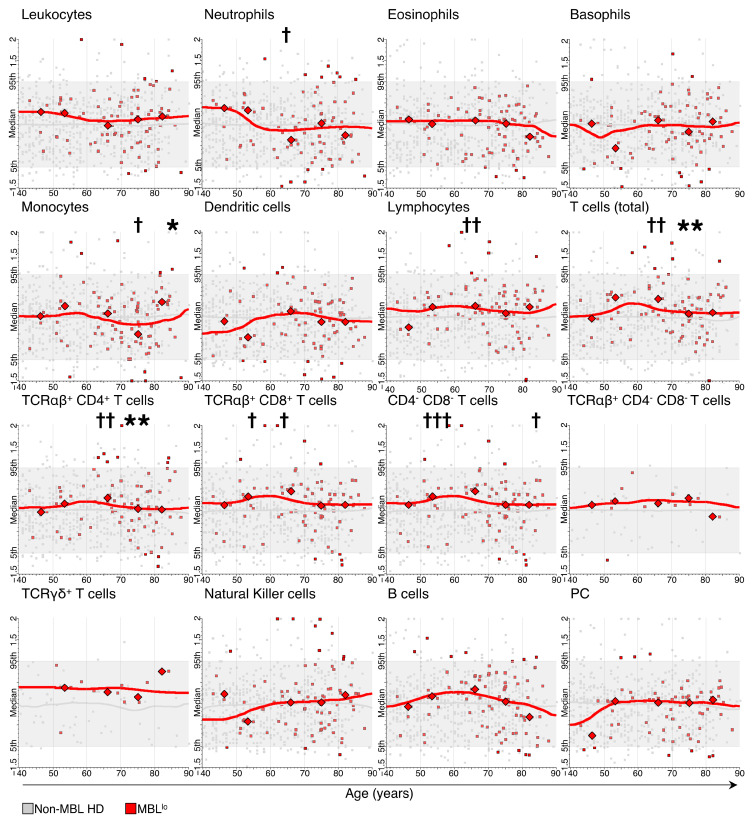
Comparison between the absolute number of the major populations of blood leukocytes identified with the LST antibody combination in MBL^lo^ vs. non-MBL healthy donors (HD) through adulthood. Grey and red dots correspond to individual non-MBL and MBL^lo^ donors, respectively. Data normalized by age is represented based on the absolute number of cells/µL for each leukocyte population analyzed, using non-MBL HD as reference. The grey horizontal box and line represent the 5th to 95th percentile range and median values observed for non-MBL HD, while the red line represents the equally weighted median value for MBL^lo^ cases. Vertical lines delineate age ranges. Statistically significant differences in the absolute number of cells found in MBL^lo^ vs. non-MBL HD per age group are depicted with crosses (*p*-value: † <0.05, †† ≤0.01, and ††† ≤0.001), while statistically significant differences in the absolute number of cells vs. the previous age group within MBL^lo^ individuals are depicted with asterisks (*p*-value: * ≤0.05 and ** ≤0.01).

Overall, consistent changes in the absolute number of circulating blood cells were observed in MBL^lo^ subjects throughout adulthood for the monocyte, T-cell (particularly at the expense of TCRαβ^+^CD4^+^ cells) and B-cell counts (Supplementary Figure S2). These included a significant increase in monocyte counts after the age of 80 y (median: 475 cells/µL vs. 288 cells/µL in the previous age group [*p* = 0.019]) (Figure 2, Supplementary Table S5 and Supplementary Figure S2). In contrast, decreasing counts of total T cells, mostly at the expense of TCRαβ^+^CD4^+^, were found among individuals aged 70–79 y vs. 60–69 y (median: 1201 T cells/µL vs. 1618 T cells/µL [*p* = 0.005] and 669 TCRαβ^+^CD4^+^ cells/µL vs. 868 TCRαβ^+^CD4^+^ cells/µL [*p* = 0.002], respectively) (Figure 2 and Supplementary Table S5 and Supplementary Figure S2).

**Figure 3:**
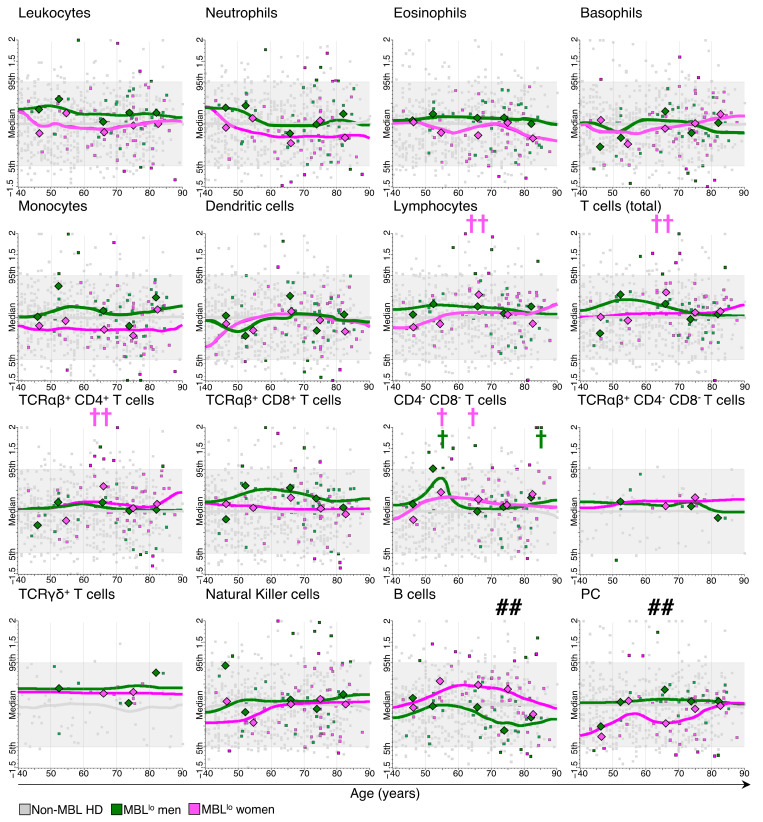
Comparison between the absolute number of the major populations of blood leukocytes identified with the LST antibody combination in MBL^lo^ vs. non-MBL healthy donors grouped by sex through adulthood. Green and magenta dots correspond to MBL^lo^ men’s and women’s cases, respectively, while (small) grey dots correspond to non-MBL healthy donors (HD). The data depicted represents the absolute number of cells/µL for the populations analyzed normalized by age, using non-MBL HD as reference. The grey horizontal box and lines represent 5th to 95th percentile and median values for non-MBL HD, while the green and magenta lines represent the equally weighted running median values for MBL^lo^ men and MBL^lo^ women, respectively. Statistically significant differences in the absolute number of cells presenting with MBL^lo^ and sex- and age-matched non-MBL HD are depicted with crosses (*p*-value: † ≤0.05 and †† ≤0.01; magenta and green crosses correspond to MBL^lo^ women and men, respectively). Statistically significant differences between women and men (within the same age group) are indicated with hashtags (*p*-value: ## <0.01).

The aforementioned increased numbers of monocytes among MBL^lo^ individuals aged ≥80 y was mostly due to the higher levels observed in men (median: 560 monocytes/µL vs. 324 monocytes/µL [*p* = 0.005] for ≥80 y vs. 70–79 y men, respectively) (Figure 3, Supplementary Table S6 and Supplementary Figure S2). Similarly, the decreased numbers of both total T cells and TCRαβ^+^CD4^+^ cells found among MBL^lo^ subjects aged >60 y reflected progressively reduced counts of both cell populations in MBL^lo^, particularly at the age of 70–79 y (median of TCRαβ^+^CD4^+^ T cells: 654 cells/µL vs. 819 cells/µL [*p* = 0.011] for 70–79 y vs. 60–69 y men, respectively) (Supplementary Table S6 and Supplementary Figure S2).

When considering sex-related differences by age ranges among MBL^lo^ subjects, despite absolute B-cell counts tending to remain higher in MBL^lo^ women (vs. MBL^lo^ men) through adulthood, similar to what is described above for non-MBL donors, these differences only reached statistical significance for subjects aged 70–79 y (median: 71 B cells/µL in men vs. 190 B cells/µL in women [*p* = 0.0045], respectively) (Figure 3 and Supplementary Table S6). In contrast, PC counts were higher among men vs. women, with such differences reaching statistical significance among MBL^lo^ individuals aged 60–69 y (median: 2.6 PC/µL in men vs. 0.81 PC/µL in women [*p* = 0.0052], respectively) (Figure 3 and Supplementary Table S6).

### 3.3. Distribution of Major Leukocyte Populations in MBL^lo^ vs. Non-MBL Subjects through Adulthood

After normalization of the absolute number of leukocyte subsets found in MBL^lo^ subjects against non-MBL donors, median cell counts for either the whole MBL^lo^ series or by age group systematically fell within the 5th–95th percentile range defined by non-MBL individuals for all leukocyte subsets (Figure 2). Nonetheless, some statistically significant different median cell counts (cell/µL) were found between MBL^lo^ and non-MBL donors for specific age ranges. Thus, neutrophil and monocyte counts were significantly lower among MBL^lo^ subjects aged 60–69 y and 70–79 y compared to their non-MBL counterparts (median: 3174 neutrophils/µL vs. 3748 neutrophils/µL [*p* = 0.017] and 288 monocytes/µL vs. 361 monocytes/µL [*p* = 0.012], respectively) (Figure 2). In contrast, significantly higher total lymphocyte and total T-cell counts, due to both greater TCRαβ^+^CD4^+^ and TCRαβ^+^CD8^+^ T-cell counts, were found in MBL^lo^ donors aged 60–69 y compared to non-MBL cases (median: 2009 lymphocytes/μL vs. 1645 lymphocytes/μL [*p* = 0.003]; 1618 T cells/μL vs. 1179 T cells/μL [*p* = 0.003]; 868 TCRαβ^+^CD4^+^ cells/μL vs. 714 TCRαβ^+^CD4^+^ cells/μL [*p* = 0.046]; and 642 TCRαβ^+^CD8^+^ cells/μL vs. 388 TCRαβ^+^CD8^+^ cells/μL [*p* = 0.014], respectively) (Figure 2). In addition, TCRαβ^+^CD8^+^ T-cell counts were also increased in MBL^lo^ cases aged 50–59 y (median: 606 cells/μL vs. 410 cells/μL for non-MBL donors [*p* = 0.039]). Similarly, TCRαβ^+^CD4^−^CD8^−^ T-cell counts were also significantly higher among MBL^lo^ individuals aged 50–59 y and ≥80 y compared to non-MBL cases (median: 126 cells/μL vs. 51 cells/μL [*p* = 0.001] and 47 cells/μL vs. 27 cells/μL [*p* = 0.011], respectively) (Figure 2).

When considering the impact of sex in the distribution of the absolute number of the different leukocyte populations here analyzed, we found that the median cell count values for both MBL^lo^ men and women systematically fell within the 5th–95th percentile range defined based on the non-MBL subjects after age-based normalization of the data. Despite this overall behavior, median total lymphocyte and T-cell counts were significantly higher among MBL^lo^ vs. non-MBL women aged 60–69 y (median: 2357 lymphocytes/μL vs. 1582 lymphocytes/μL, respectively [*p* = 0.002]) (Figure 3), mostly due to increased numbers of TCRαβ^+^CD4^+^ T cells (median: 1040 TCRαβ^+^CD4^+^ T cells/μL in MBL^lo^ vs. 706 TCRαβ^+^CD4^+^ T cells/μL in non-MBL cases [*p* = 0.009]) (Figure 3) and, to a lesser extent, also to CD4^−^CD8^−^ T cells (median: 70 CD4^−^CD8^−^ T cells/μL in MBL^lo^ vs. 39 CD4^−^CD8^−^ T cells/μL in non-MBL cases, [*p* = 0.012]) (Figure 3), both leading to an overall increase in the number of total T cells in MBL^lo^ vs. non-MBL women (median: 1902 T cells/μL vs. 1116 T cells/μL, respectively [*p* = 0.005]) (Figure 3). Of note, CD4^−^CD8^−^ T-cell counts were also increased in both MBL^lo^ women and MBL^lo^ men aged 50–59 y (median: 89 CD4^−^CD8^−^ T cells/μL vs. 40 CD4^−^CD8^−^ T cells/μL [*p* = 0.027] and 149 CD4^−^CD8^−^ T cells/μL vs. 41 CD4^−^CD8^−^ T cells/μL [*p* = 0.018], respectively) (Figure 3) and among men aged ≥80 y compared to non-MBL men of the same age (median: 46 CD4^−^CD8^−^ T cells/μL vs. 23 CD4^−^CD8^−^ T cells/μL [*p* = 0.013]) (Figure 3).

## 4. Discussion

Adequate interpretation of the kinetics in blood of the different immune cell populations in various conditions (e.g., aging, immunodeficiency, inflammation and cancer) requires robust reference ranges obtained through standardized and validated techniques in large cohorts of healthy individuals of different age, sex and ethnicity. For this purpose, standardized and validated flow cytometry protocols, antibody panels, and data acquisition and analysis strategies were applied in this study for high-sensitivity detection and enumeration of the major populations of blood leukocytes [28,29,30,33]. To our knowledge, this is the first report that provides (normal) blood reference ranges, matched per age and sex, for the major populations of leukocytes circulating in blood as identified by high-sensitivity and standardized flow cytometry, based on the largest cohort of adult Caucasians, in which the absence (non-MBL HD) vs. presence in blood of small B-cell clones with a CLL/SLL-like phenotype (MBL^lo^) is also taken into account [28,29,30,33].

Overall, our results support and extend previous findings on the distribution of absolute leukocyte subsets in blood of non-MBL HD [8,9,12,13]. Overall, a tendency towards progressively (non-significant) lower WBC counts was observed throughout adulthood with repeatedly higher WBC counts among middle-aged men vs. women. As expected, most normal ranges here reported for total leukocytes were similar to those previously obtained through the use of hematologic cell counters [6,34].

Among innate immune cells, progressively higher eosinophil counts were observed through adulthood. Of note, the number of eosinophils was higher among middle-aged men than women, reaching similar values after the age of 70. Previous reports in which five-part differential counts obtained in certified hematological cell analyzers were used showed similar kinetics for eosinophils in blood as those described here [35,36,37]. Interestingly, middle-aged (40–69 y) men constantly showed higher monocyte counts than women. Despite this, no clear trend was observed in the number of blood monocytes throughout adulthood, in line with recently reported data in a more limited cohort (*n* = 164) of healthy donors categorized by age but not by sex [9].

Overall, total lymphocyte, T-cell, B-cell and plasma cell counts showed a tendency toward progressively decreased counts with age, with highly reduced counts in men aged 70–79 y, particularly of TCRαβ^+^CD4^+^ and TCRαβ^+^CD8^+^ T cells. These results are in line with previous observations [4,6,12,38] (in Caucasian populations [6,8,12]), which contrasts to the more stable cell numbers for these cell populations reported in Asian (Korean) populations [4]. In line with these observations, different WBC counts have been reported between Caucasian and Afro-American populations in the United States of America [34], which emphasizes the relevance of ethnicity when defining normal values and disease-associated altered counts.

Our study widened its scope beyond the most represented and thus better-known T-cell populations, providing information also for less frequent T-cell subsets, such as CD4^−^CD8^−^ and TCRγδ^+^ T-cell subsets. Interestingly, total T CD4^−^CD8^−^ cells were found to be decreased both at the age of 40–49 y and in individuals ≥80 y. This same trend was observed for both TCRαβ^+^CD4^−^CD8^−^ and TCRγδ^+^ T cells, in line with data available in the literature [38]. In contrast to T- and B-cell counts, our results show that NK-cell counts gradually increased with age, being higher among middle-aged men than women. These results are in line with those of a few previous reports carried out in smaller cohorts [4,6].

The mechanisms by which aging induces immune-related changes—reflected in the distribution of leukocyte subsets in the blood—are not yet fully understood. Despite this, a progressive pro-inflammatory state throughout adulthood, which would promote myelopoiesis, paralleled by an immunosuppressive microenvironment affecting both T- and B-cell production and functionality, have been reported [39]. These findings are in line with the observation of a shift of bone marrow hematopoietic stem cells during aging toward myeloid-cell production, while the ability of stem cells to differentiate into B-cell progenitors is compromised [40]. However, it should be noted that the effect of aging on leukocyte kinetics in blood is independent of the presence of small B-cell clones, as demonstrated here for the first time for non-MBL and MBL^lo^ subjects.

In addition to the fluctuations in the absolute number of the major leukocyte populations attributable to aging, non-MBL middle-aged men systematically showed higher counts of myeloid cells (except for dendritic cells) than women. By contrast, individuals aged ≥70 y showed lower T-cell population and B-cell counts in men than in women. Overall, these results point to the relevance of sex in the distribution of leukocyte populations in blood, with a tendency towards more prominent and earlier aging-associated changes in men than women. The precise mechanisms underlying the above reported differences between sexes are not well understood. Despite this, it has been hypothesized that the level of sex hormones might have an impact on the distribution of the leukocyte populations and the strength of inflammatory processes in men vs. women, independently of the ethnicity [35,41,42]. In this regard, the incidence of autoimmune- and inflammatory-related diseases (e.g., coronary heart disease) varies in men vs. women aged <50–55 y, while such differences tend to vanish after menopausal age, when the hormonal profile of women drastically changes [43]. As such, the influence of sex hormones in the distribution of blood cell subsets at different age ranges through life still remain to be deeply investigated.

Overall, median cell-counts for all blood leukocyte populations investigated in MBL^lo^ subjects were always within the 5th–95th percentile range defined by non-MBL HD values. Despite this, independently of the age group, the median overall number of circulating lymphocytes in MBL^lo^ individuals aged 60–69 y was significantly higher than that of non-MBL subjects, mostly due to the increase of TCRαβ^+^CD4^+^ and TCRαβ^+^CD8^+^ T-cell counts. Similarly, Faria-Moss et al. found significantly higher T-cell counts in blood in a series of (Asian) MBL^lo^ cases, in which TCRαβ^+^CD4^+^- and TCRαβ^+^CD8^+^-cell counts followed the same trend [20]. In line with these findings, another report described an overall increase of CD8^+^ T-cell counts among MBL^lo^ cases compared to HD, but no differences were found when corrected by age [22]. This later report also described overall reduced B-cell counts in blood among MBL^lo^ subjects, mostly at the expense of the immature and naïve B-cell populations; however, for the CD8^+^ T-cell counts, these differences did not stand after correction by age [22], concurring with previous findings of our group in a more limited cohort of donors (27 CLL-like MBL^lo^ vs. 40 non-MBL HD) [23] and the results reported here. In turn, the differences here observed between MBL^lo^ men and women paralleled those found among non-MBL subjects for B lymphocytes (higher levels in oldest women) and PC (higher levels among men vs. women), although these differences were more pronounced in MBL^lo^ subjects than in non-MBL controls, suggesting a more pronounced B-cell-associated aging profile in the former group [35,41,42].

Most interestingly, here we report for the first time a significant increase in CD4^−^CD8^−^ T-cell counts among CLL-like MBL^lo^ individuals compared to non-MBL subjects. Overall, CD4^−^CD8^−^ T cells have been long associated with cytotoxic and regulatory functions [44], and they are known to be increased in some cancers, including CLL [45], particularly at the infiltrated tumor sites [46,47,48], where they are believed to play an antitumoral role. In this regard, higher CD4^−^CD8^−^ T-cell counts might reflect the occurrence of underlying (oligo) clonal expansions of innate T cells in the context of an emerging MBL^lo^ clone. Interestingly, the median number of CD4^−^CD8^−^ T cells was found to be consistently higher in MBL^lo^ vs. non-MBL donors, with differences reaching statistical significance among individuals aged 50–59 y and ≥80 y, which could be due to the overall increased cell counts observed in the whole cohort among individuals aged 50–59 y and the impact of sex-associated differences in the ≥80 y group.

A major strength of the present study relies on its high sensitivity and reproducibility thanks to the use of highly standardized and optimized flow cytometry protocols and panels for an in-depth dissection of up to 16 different leukocyte populations in blood. In addition, this study is the first in which individuals with MBL^lo^, known to be associated with some alterations in the distribution of specific populations of circulating leukocytes, were specifically identified and separately considered [6,7,10,11,31,38]. However, the present study has some limitations. Thus, since the vast majority of the population living in the Salamanca area is Caucasian, no individuals from other ethnicities were studied, pointing out the need to extend this analysis to other ethnic populations and geographical areas to shed light on the genetic and environmental factors that influence the distribution of the different leukocyte populations in blood and to establish more accurate reference ranges. In turn, despite here the presence of MBL^lo^ clones was systematically investigated, there is a clear imbalanced number of MBL^lo^ vs. non-MBL subjects, particularly among the youngest age groups (18–49 y). This results from the overall frequency of MBL^lo^ in the general population (3.5–17%) whose prevalence increases with age [14,15,16,17,18,19,20,21].

## 5. Conclusions

In summary, here we define for the first time normal reference ranges for the major populations of blood-circulating leukocytes using a standardized and validated high-sensitive flow cytometry technique based on a large cohort of adult Caucasians from the general population in whom age, sex and the presence of small clonal B-cell populations were considered for a more robust definition of reference values. The availability of these new reference ranges for the major populations of blood leukocytes is a prerequisite for the identification and characterization of potentially altered immune profiles in different physiological and clinical settings.

## Data Availability

Not applicable.

## Supplementary Materials

The following supporting information can be downloaded at: https://www.mdpi.com/article/10.3390/cancers15010058/s1, Supplementary methods File S1: sample preparation and staining and Data normalization and plotting using MIDAS (v2.0.5.d); Table S1: Age distribution of the healthy adults that participated in the study according to sex and the presence vs. absence of MBL; Table S2: Combinations of monoclonal antibodies and fluorochrome-conjugated reagents included in the different EuroFlow™ LST formats used in this study; Table S3: Distribution of the major populations of blood leukocytes identified with the EuroFlow LST antibody combination in adult healthy donors grouped by age; Table S4: Distribution of the major populations of blood leukocytes identified with the EuroFlow LST antibody combination in adult heathy donors grouped by age and sex; Table S5: Distribution of the major populations of blood leukocytes identified with the EuroFlow LST antibody combination in MBL^lo^ individuals grouped by age; Table S6: Distribution of the major populations of blood leukocytes identified with the EuroFlow LST antibody combination in MBL^lo^ subjects grouped by age and sex; Table S7: List of primary care physicians of the Primary Health Care Group of Salamanca for the Study of MBL that have participated in the recruitment of donors. Figure S1: Summary of the statistically significant differences identified in the absolute cell counts for the major populations of blood leukocytes identified with the EuroFlow LST antibody combination in non-MBL HDs through adulthood; Figure S2: Summary of the statistically significant differences identified in the absolute cell counts for the major populations of blood leukocytes identified with the EuroFlow LST antibody combination in MBL^lo^ through adulthood.
