# Supplementary material for: Age- and Sex-Matched Normal Leukocyte Subset Ranges in the General Population Defined with the EuroFlow Lymphocyte Screening Tube (LST) for Monoclonal B-Cell Lymphocytosis (MBL) vs. Non-MBL Subjects
Source: Cancers (Basel). 2022 Dec 22;15(1):58. doi: 10.3390/cancers15010058 (PMC9817826; doi:10.3390/cancers15010058)
Supplement: Supplementary file 1 [file cancers-15-00058-s001.zip › cancers-2023962-supplementary.pdf]

SUPPLEMENTARY MATERIALS

SUPPLEMENTARY METHODS File S1

Sample preparation and staining. To reach a sensitivity of $\leq 10^{-5}$ (i.e., identification of at least 1 cell of interest in 100,000 total leukocytes) data on a minimum of 5×10^6 leukocytes was collected. For this purpose, 800 µL of blood were processed per sample following the stain-lyse-and-then-wash protocol recommended by EuroFlow for the staining of blood samples with the LST antibody combination. Before the staining, 200 µL aliquots of blood were transferred to individual tubes and washed twice with 2 mL of phosphate-buffered saline (PBS). Washed blood was incubated for 30 min at room temperature (RT) with the appropriate volume of fluorochrome-conjugated antibodies or placed into OneFlow™ LST (BD Biosciences, San Jose, CA) tubes. Subsequently, 2 mL of 1xFACSLysing solution (BD Biosciences; San Jose, CA) were added, properly mixed, and incubated for another 10 min at RT (in the darkness). After a centrifugation step (5 min at 540g), the supernatant was discarded, and the cell pellet resuspended in 2 mL PBS containing 0.2% bovine serum albumin (BSA), 0.1% sodium azide (NaAz) and 2 mM EDTA (pH = 7.4) (from now on "PBS-BSA"). Following an additional centrifugation step (5 min at 540g), the supernatant was discarded, and the cell pellet resuspended in 250 µL PBS-BSA. Cells were immediately acquired after sample preparation was completed or they were stored at 4-8ºC for <1 hour.

Data normalization and plotting using MIDAS (v2.0.5.d) (Cytognos SL, Salamanca, Spain)**:** Age-based normalization of the cell counts for the different leukocyte populations found in each MBL^{lo} individual was performed against non-MBL donors of the same age using the following formula:

- If the value of a given population ($v(i)$) is below the median of the reference subgroup (Bi)(i.e., $v(i) < median(v(Bi))$:

$$n(i) = \frac{v(i) - median(v(Bi))}{median(v(Bi)) - percentile5(v(Bi))}$$

- If the value of a given population ($v(i)$) is above the median of the reference subgroup (Bi) (i.e., $v(i) \geq median(v(Bi))$:

$$n(i) = \frac{v(i) - median(v(Bi))}{percentile95(v(Bi)) - median(v(Bi))}$$

Where the symbols and functions (by order of appearance) are defined as follows: **n(*i*):** normalized value for a given individual "i"; **v(*i*):** the actual value of a given cell population (cell/µL) of the case to be normalized; **B*i*:** the reference subgroup of all those non-MBL HD with an age ranging from <10y to >10y of the age of each given individual "i".

SUPPLEMENTARY TABLES:

TABLE S1. Age distribution of the healthy adults that participated in the study according to sex and the presence vs. absence of MBL.

	18–39y	40–49y	50–59y	60–69y	70–79y	≥80y	Total
**Whole cohort**	32 (23–39)<br>*n* = 23	44 (40–49)<br>*n* = 154	54 (50–59)<br>*n* = 152	64 (60–69)<br>*n* = 136	74 (70–79)<br>*n* = 178	82 (80–97)<br>*n* = 63	61 (23–97)<br>*n* = 706
Men	34 (23–37)<br>*n* = 9	45 (40–49)<br>*n* = 64	54 (50–59)<br>*n* = 74	64 (60–69)<br>*n* = 67	74 (70–79)<br>*n* = 82	83 (80–89)<br>*n* = 24	61 (23–89)<br>*n* = 320
Women	32 (25–39)<br>*n* = 14	44 (40–49)<br>*n* = 90	55 (50–59)<br>*n* = 78	65 (60–69)<br>*n* = 69	74 (70–79)<br>*n* = 96	82 (80–97)<br>*n* = 39	62 (25–97)<br>*n* = 386
**Non-MBL HD**	32 (23–39)<br>*n* = 23	44 (40–49)<br>*n* = 149	55 (50–59)<br>*n* = 140	64 (60–69)<br>*n* = 119	74 (70–79)<br>*n* = 146	82 (80–97)<br>*n* = 45	59 (23–97)<br>*n* = 622
Men	34 (23–37)<br>*n* = 9	45 (40–49)<br>*n* = 62	54 (50–59)<br>*n* = 68	64 (60–69)<br>*n* = 61	74 (70–79)<br>*n* = 69	83 (80–89)<br>*n* = 17	60 (23–89)<br>*n* = 286
Women	32 (25–39)<br>*n* = 14	44 (40–49)<br>*n* = 87	55 (50–59)<br>*n* = 72	65 (60–69)<br>*n* = 58	73 (70–79)<br>*n* = 77	82 (80–97)<br>*n* = 28	58 (25–97)<br>*n* = 336
**MBL^{lo}**	NA	46 (43–48)<br>*n* = 5	53 (51–59)<br>*n* = 12	66 (61–69)<br>*n* = 17	75 (70–79)<br>*n* = 32	83 (80–93)<br>*n* = 18	73 (43–93)<br>*n* = 84
Men	NA	46 (44–48)<br>*n* = 2	52 (51–58)<br>*n* = 6	66 (64–68)<br>*n* = 6	74 (70–79)<br>*n* = 13	82 (80–85)<br>*n* = 7	72 (44–85)<br>*n* = 34
Women	NA	46 (43–47)<br>*n* = 3	55 (52–59)<br>*n* = 6	66 (61–69)<br>*n* = 11	75 (70–79)<br>*n* = 19	84 (80–93)<br>*n* = 11	73 (43–93)<br>*n* = 50

Results expressed as median (minimum-maximum) years of age, and number (*n*) of cases within the corresponding subgroup. No statistically significant differences were observed between non-MBL HD and MBL^{lo} cases when classified by age group and gender. Abbreviations (in alphabetical order): **HD:** healthy donor; **MBL^{lo}:** low-count monoclonal B-cell lymphocytosis; **n**: number; **NA:** not applicable, as no MBL^{lo} cases were detected for this age group; **y:** years.

TABLE S2. Combinations of monoclonal antibodies and fluorochrome conjugated reagents included in the different EuroFlow™ LST formats used in this study.

A. LST format without anti-TCRγδ (applied in samples collected between 2007–2011):

Fluorochrome	PacB	PacO	FITC	PE	PerCPCy5.5	PECy7	APC	AF700
**Marker**	CD20<br>CD4	CD45	CD8<br>λ	CD56<br>κ	CD5	CD19	CD3	CD38
**Clone**	2H7<br>RPA-T4	HI30	UCHT4<br>polyclonal	C5.9<br>polyclonal	L200	HI10a	UCHT2	HB7
**Manufacturer**	eBioscience<br>BD	Invitrogen	BD<br>Dako	BD<br>Dako	BD	BC	BD	BD

B. EuroFlow™ LST (applied in samples collected between 2012–2017):

Fluorochrome	PacB	OC515	FITC	PE	PerCPCy5.5	PECy7	APC	APCH7
**Marker**	CD20<br>CD4	CD45	CD8<br>λ	CD56<br>κ	CD5	CD19<br>TCRγδ	CD3	CD38
**Clone**	2H7<br>RPA-T4	HI30	UCHT4<br>polyclonal	C5.9<br>polyclonal	L200	HI10a<br>11F2	UCHT2	HB7
**Manufacturer**	eBioscience<br>BD	Cytognos	Cytognos	Cytognos	BD	BC<br>BD	BD	BD

C. BD OneFlow EuroFlow™ LST lyophilized tube (BD Biosciences, San Jose, CA; Ref: 655619) (applied in samples collected between 2018–2019):

Fluorochrome	PacB	PacO	FITC	PE	PerCPCy5.5	PECy7	APC	APCH7
**Marker**	CD20<br>CD4	CD45	CD8<br>λ	CD56<br>κ	CD5	CD19<br>TCRγδ	CD3	CD38
**Clone**	L27<br>SK3	2D1	SK1<br>1-155-2	My31<br>TB28-2	L17F12	SJ25-C1<br>11F2	SK7	HB7
**Manufacturer**	BD							

Abbreviations for fluorochromes (alphabetical order): **AF700:** alexa fluor® 700; **APC:** allophycocyanine; **APCH7:** allophycocyanine-hilite® 7; **FITC:** fluorescein isothiocyanate; **OC515:** Orange Cytognos 515; **PacB:** Pacific Blue™; **PacO:** Pacific Orange™; **PE:** phycoerythrin; **PECy7:** phycoerythrin-cyanine7; **PerCPCy5.5:** peridinin chlorophyll protein-complex cyanine 5.5.
Other abbreviations (alphabetical order): **BC:** Beckman Coulter, Brea, CA; **BD:** Becton Dickinson Biosciences, San José, CA; **LST:** Lymphocyte Screening Tube.

TABLE S3. Distribution of the major populations of blood leukocytes identified with the EuroFlow LST antibody combination in adult healthy donors (HD) grouped by age.

Populations	Age group					
	**18–39y (*n* = 23)**	**40–49y (*n* = 149)**	**50–59y (*n* = 140)**	**60–69y (*n* = 119)**	**70–79y (*n* = 146)**	**≥80y (*n* = 45)**
Leukocytes	5890 [4040-7980] (3630-9200)	6070 [4080-10410] (3270-12390)	6030 [3850-9690] (2920-11400)	6210 [4160-9120] (3370-12140)	5900 [3910-8890] (3510-11190)	6060 [4600-9410] (4130-14000)
Neutrophils	3616 [2204-5251] (2057-6781)	3778 [2314-6954] (1764-8073)	3578 [2226-6468] (1563-8071)	3748 [2311-6200] (1758-7812)	3672 [2096-5665] (1672-7658)	3739 [2624-6843] (2334-8542)
Eosinophils	104 [38-713] (34-717)	**176 ** [49-511] (10-834)**	151 [51-445] (3.7-784)	167 [51-520] (12-876)	160 [46-425] (3.7-663)	196 [60-440] (32-733)
Basophils	29 [12-107] (1.7-406)	39 [15-67] (2.2-222)	37 [12-88] (<0.10-135)	41 [12-85] (<0.10-102)	39 [10-83] (<0.10-130)	37 [19-83] (<0.10-101)
Monocytes	396 [195-531] (194-559)	363 [196-754] (109-1163)	319 [146-694] (82-1189)	359 [180-746] (25-1243)	361 [175-708] (38-977)	373 [220-636] (215-1035)
Dendritic cells	13 [3-28] (2.5-41)	17 [3.9-47] (1.4-67)	15 [4.0-36] (0.85-118)	15 [5.0-34] (<0.10-93)	16 [4.3-46] (2.0-77)	14 [4.7-36] (1.7-39)
Lymphocytes	1746 [1069-2453] (881-2564)	1682 [925-3476] (407-5182)	1757 [936-3040] (610-3446)	1645 [828-3036] (691-4130)	1616 [891-3035] (627-4125)	1613 [938-2947] (767-5947)
T cells	1290 [806-1929] (525-2190)	1283 [663-2497] (371-4458)	1276 [629-2228] (504-2829)	1179 [603-2397] (524-2727)	1098 [594-2285] (435-2986)	1171 [672-2129] (616-5298)
TCRαβ $CD4^+$	764 [418-1256] (363-1502)	705 [318-1524] (224-3054)	718 [369-1421] (106-1732)	714 [348-1290] (125-1682)	652 [310-1257] (192-1559)	594 [276-1379] (158-2159)
TCRαβ $CD8^+$	384 [228-647] (126-695)	397 [187-978] (92-2199)	410 [170-1030] (124-1486)	388 [139-890] (65-1483)	362 [136-861] (78-1939)	409 [175-1277] (109-1939)
$CD4^-$ $CD8^-$	79 [36-243] (25-267)	**51 ** [14-175] (4.6-291)**	40 [11-147] (1.9-495)	39 [8.3-134] (3.3-252)	43 [9.3-155] (4.0-354)	**27 ** [6.6-88] (2.7-133)**
TCRαβ $CD4^-$ $CD8^-$	11 [5.8-24] (2.0-36)	18 [5.2-117] (5.2-117)	17 [4.6-28] (4.6-28)	15 [6.1-54] (6.1-54)	22 [7.6-37] (7.6-37)	15 [12-67] (12-67)
TCRγδ	63 [23-226] (19-257)	51 [6.5-208] (6.5-208)	40 [13-185] (13-185)	37 [22-226] (22-226)	54 [14-317] (14-317)	22 [13-119] (13-119)
NK cells	235 [68-426] (42-460)	228 [78-640] (29-875)	257 [79-688] (6.8-932)	286 [89-736] (58-1153)	318 [109-804] (56-1273)	286 [66-778] (39-965)
B cells	157 [88-249] (77-271)	155 [64-498] (8.1-678)	159 [55-353] (29-619)	140 [55-320] (19-411)	**121 * [44-298] (19-776)**	**102 * [34-223] (16-300)**
PC	1.7 [0.53-7.0] (0.14-8.7)	1.7 [0.46-7.9] (0.19-23)	**1.2 ** [0.35-5.8] (<0.10-18)**	1.4 [0.25-4.9] (0.10-18)	1.2 [0.21-5.2] (0.13-13)	0.94 [0.26-5.3] (<0.10-9.9)

Results expressed as median [5th-95th percentile] (minimum-maximum) absolute cell counts /µL of blood. *p ≤0.05 and **p ≤0.01 versus the previous age group are highlighted in **bold**. Abbreviations in alphabetical order: **n:** number; **NK:** natural killer; **PC:** plasma cell; **TCR:** T-cell receptor; **y:** year

TABLE S4. Distribution of the major populations of blood leukocytes identified with the EuroFlow LST antibody combination in adult heathy donors grouped by age and sex.

	Age group											
	18-39y (*n* = 23)		40-49y (*n* = 149)		50-59y (*n* = 140)		60-69y (*n* = 119)		70-79y (*n* = 146)		≥80y (*n* = 45)	
Cell population	M (*n* = 9)	W (*n* = 14)	M (*n* = 62)	W (*n* = 87)	M (*n* = 68)	W (*n* = 72)	M (*n* = 61)	W (*n* = 58)	M (*n* = 69)	W (*n* = 77)	M (*n* = 17)	W (*n* = 28)
Leukocytes	5660<br>[4040-7700]<br>(4040-7700)	6725<br>[3630-9200]<br>(3630-9200)	6690<br>[3720-10630]<br>(3520-12390)	5710 #<br>[4200-9820]<br>(3270-11070)	6425<br>[3990-9750]<br>(3470-11400)	5765 #<br>[3600-8350]<br>(2920-10660)	6470<br>[4750-9450]<br>(3530-12140)	5980 #<br>[4030-8100]<br>(3370-8870)	6100<br>[3980-8570]<br>(3510-10420)	5820<br>[3840-9250]<br>(3610-11190)	6060<br>[4130-8800]<br>(4130-8800)	6080<br>[4680-11240]<br>(4500-14000)
Neutrophils	3572<br>[2317-5056]<br>(2317-5056)	4029<br>[2057-6781]<br>(2057-6781)	3895<br>[2386-7281]<br>(1764-8073)	3686<br>[2286-6571]<br>(1779-7653)	3696<br>[2252-6509]<br>(1769-8071)	3330 #<br>[2200-5662]<br>(1563-7334)	3766<br>[2382-6200]<br>(1758-7603)	3646<br>[2294-5959]<br>(1980-7812)	3819<br>[2527-5549]<br>(1672-7034)	3525<br>[2094-6105]<br>(1697-7658)	3739<br>[2334-6477]<br>(2334-6477)	3744<br>[2678-7359]<br>(2624-8542)
Eosinophils	92<br>[34-717]<br>(34-717)	111<br>[38-713]<br>(38-713)	206 **<br>[67-531]<br>(31-834)	128 ##<br>[46-417]<br>(10-752)	187<br>[68-455]<br>(43-784)	116 ###<br>[43-420]<br>(3.7-557)	173<br>[52-548]<br>(24-799)	157<br>[40-503]<br>(12-876)	161<br>[53-344]<br>(16-663)	157<br>[45-445]<br>(3.7-512)	167<br>[57-524]<br>(57-524)	197<br>[60-397]<br>(32-733)
Basophils	29<br>[15-107]<br>(15-107)	32<br>[1.7-406]<br>(1.7-406)	45<br>[15-86]<br>(2.2-222)	34 ##<br>[17-64]<br>(6.9-111)	41<br>[13-99]<br>(<0.1-135)	36<br>[11-82]<br>(6.1-117)	44<br>[15-85]<br>(<0.1-100)	41<br>[5.1-75]<br>(0-102)	36<br>[10-74]<br>(7.4-113)	40<br>[8.3-83]<br>(<0.1-130)	26<br>[20-83]<br>(20-83)	39<br>[7-83]<br>(<0.1-101)
Monocytes	413<br>[292-559]<br>(292-559)	347<br>[194-524]<br>(194-524)	409<br>[246-795]<br>(109-1163)	344 ##<br>[182-637]<br>(111-782)	353<br>[180-694]<br>(106-1189)	302 #<br>[146-632]<br>(82-916)	385<br>[196-772]<br>(25-1243)	322 #<br>[180-643]<br>(129-795)	356<br>[181-724]<br>(81-885)	364<br>[153-690]<br>(39-977)	403<br>[215-580]<br>(215-580)	371<br>[241-1029]<br>(220-1035)
Dendritic cells	13<br>[2.5-41]<br>(2.5-41)	14<br>[4.7-28]<br>(4.7-28)	18<br>[3.9-40]<br>(2.7-48)	16<br>[3.5-49]<br>(1.4-67)	15<br>[3.4-35]<br>(1.6-118)	16<br>[4.1-37]<br>(0.85-72)	15<br>[5.2-32]<br>(0.61-50)	14<br>[5.0-42]<br>(<0.1-93)	16<br>[3.3-49]<br>(2.0-72)	15<br>[4.8-46]<br>(2.0-77)	14<br>[4.7-31]<br>(4.7-31)	14<br>[3.2-36]<br>(1.7-39)
Lymphocytes	1626<br>[881-2182]<br>(881-2182)	1782<br>[1069-2564]<br>(1069-2564)	1756<br>[969-3524]<br>(407-4137)	1604<br>[925-3324]<br>(652-5182)	1778<br>[1044-3083]<br>(610-3446)	1654<br>[920-2768]<br>(739-3287)	1743<br>[1000-3089]<br>(691-4130)	1582 #<br>[774-2568]<br>(733-3701)	1446 *<br>[903-3025]<br>(627-3745)	1796<br>[881-3161]<br>(742-4125)	1379<br>[922-3098]<br>(922-309)	1659<br>[949-2947]<br>(767-5947)
T cells	1205<br>[525-1708]<br>(525-1708)	1330<br>[806-2190]<br>(806-2190)	1326<br>[663-2562]<br>(371-3228)	1242<br>[687-2320]<br>(509-4458)	1251<br>[615-2468]<br>(504-2829)	1322<br>[632-2178]<br>(531-2517)	1301<br>[695-2480]<br>(541-2727)	1116<br>[585-1917]<br>(524-2528)	972 **<br>[588-2100]<br>(435-2986)	1192<br>[594-2315]<br>(466-2951)	951<br>[672-2129]<br>(672-2129)	1326<br>[667-2293]<br>(616-5298)
TCRαβ $CD4^+$	655<br>[363-851]<br>(363-851)	813<br>[418-1502]<br>(418-1502)	700<br>[296-1580]<br>(250-1994)	708<br>[338-1500]<br>(224-3054)	694<br>[319-1357]<br>(193-1732)	754<br>[453-1454]<br>(106-1661)	717<br>[393-1265]<br>(304-1682)	706<br>[308-1299]<br>(125-1659)	578 *<br>[271-1249]<br>(192-1559)	717 #<br>[337-1300]<br>(263-1517)	493 *<br>[158-806]<br>(158-806)	646 ##<br>[356-1561]<br>(346-2159)
TCRαβ $CD8^+$	421<br>[126-647]<br>(126-647)	370<br>[229-695]<br>(229-695)	421<br>[197-1064]<br>(92-2199)	385<br>[187-960]<br>(126-1317)	411<br>[146-1072]<br>(124-1486)	405<br>[185-718]<br>(127-1323)	429<br>[193-1281]<br>(65-1483)	374 #<br>[132-719]<br>(113-890)	344 **<br>[133-850]<br>(78-1770)	362<br>[136-948]<br>(97-1939)	409<br>[196-1337]<br>(196-1337)	410<br>[139-1221]<br>(109-1939)
$CD4^-$ $CD8^-$	70<br>[25-243]<br>(25-243)	87<br>[36-267]<br>(36-267)	55<br>[14-175]<br>(8.0-218)	48 **<br>[15-143]<br>(4.6-291)	41<br>[13-195]<br>(8.0-495)	40<br>[8.0-142]<br>(1.9-219)	45<br>[11-137]<br>(3.3-252)	39<br>[7.0-123]<br>(5.0-136)	39<br>[9.1-150]<br>(4.9-354)	44<br>[9.3-172]<br>(4.0-212)	23 **<br>[2.7-55]<br>(2.7-55)	32<br>[7.3-89]<br>(6.4-133)
TCRαβ $CD4^-$ $CD8^-$	9.4<br>[5.8-23]<br>(5.8-23)	12<br>[2.0-36]<br>(2.0-36)	26<br>[10-38]<br>(10-38)	16<br>[5.2-117]<br>(5.2-117)	17<br>[4.6-27]<br>(4.6-27)	16<br>[8.2-28]<br>(8.2-28)	21<br>[6.1-54]<br>(6.1-54)	14<br>[6.5-16]<br>(6.5-16)	14<br>[8.3-37]<br>(8.3-37)	32<br>[7.6-36]<br>(7.6-36)	NAv	15<br>[12-67]<br>(12-67)
TCRγδ	59<br>[19-226]<br>(19-226)	73<br>[23-257]<br>(23-257)	79<br>[6.5-208]<br>(6.5-208)	38<br>[8.3-174]<br>(8.3-174)	75<br>[14-185]<br>(14-185)	32<br>[13-126]<br>(13-126)	62<br>[22-226]<br>(22-226)	37<br>[25-102]<br>(25-102)	48<br>[14-317]<br>(14-317)	109<br>[17-122]<br>(17-122)	NAv	22<br>[13-119]<br>(13-119)
NK cells	235<br>[42-426]<br>(42-426)	228<br>[68-460]<br>(68-460)	287<br>[93-726]<br>(29-875)	200 #<br>[72-506]<br>(61-785)	301<br>[133-780]<br>(77-932)	225 #<br>[67-516]<br>(6.8-595)	347<br>[112-736]<br>(69-1153)	252 #<br>[88-545]<br>(58-950)	322<br>[135-814]<br>(63-1215)	315<br>[79-804]<br>(56-1273)	286<br>[39-876]<br>(39-876)	279<br>[74-750]<br>(65-965)
B cells	147<br>[88-271]<br>(88-271)	161<br>[77-249]<br>(77-249)	163<br>[64-498]<br>(8.1-645)	151<br>[64-408]<br>(41-678)	159<br>[52-343]<br>(29-416)	158<br>[62-363]<br>(35-619)	144<br>[60-320]<br>(19-358)	139<br>[55-317]<br>(34-411)	104 **<br>[36-252]<br>(19-392)	132 #<br>[54-337]<br>(41-776)	83<br>[34-213]<br>(34-213)	110<br>[38-285]<br>(16-300)
PC	2.2<br>[0.53-8.7]<br>(0.53-8.7)	1.3<br>[0.14-7.0]<br>(0.14-7.0)	1.7<br>[0.49-8.1]<br>(0.35-23)	1.8<br>[0.42-7.9]<br>(0.19-13)	1.4<br>[0.46-6.3]<br>(0.13-12)	1.1<br>[0.24-4.8]<br>(0-18)	1.5<br>[0.23-4.4]<br>(0.10-11)	1.3<br>[0.33-7.1]<br>(0.21-18)	1.2<br>[0.17-5.5]<br>(0.13-13)	1.2<br>[0.28-5.0]<br>(0.17-11)	0.98<br>[0.03-1.6]<br>(0.03-1.6)	0.92<br>[0.31-6.9]<br>(0.26-9.9)

Results expressed as median [5th-95th percentiles] (range) absolute cell counts /μL of blood. The absolute cell count (/μL) of blood was compared with those of the preceding age group (*p <0.05, and **p ≤0.01 vs. the corresponding sex of the previous age group, highlighted in **bold**) and by gender within the same age group ($^{\#}p$ <0.05 and $^{\#\#}p$ ≤0.01 male vs. female; depicted in *italics* and in blue), respectively. Abbreviations in alphabetical order: **LST:** Lymphocyte Screening Tube; **M:** men; **n:** number; **NAv:** not available; **NK:** natural killer; **PC:** plasma cells; **TCR:** T-cell receptor; **W:** women; **y:** years.

TABLE S5. Distribution of the major populations of blood leukocytes identified with the EuroFlow LST antibody combination in MBL^lo individuals grouped by age.

Populations	Age group<br>**40–49y (*n* = 5)**	**50–59y (*n* = 12)**	**60–69y (*n* = 17)**	**70–79y (*n* = 32)**	**≥80y (*n* = 18)**
Leukocytes	7360 [4650–8540]	7110 [4700–13000]	6070 [4770–8470]	6310 [3770–9400]	6565 [3850–10140]
Neutrophils	4821 [2502–5605]	4598 [2925–7887]	3174 [1681–4168]	3756 [2154–6429]	3333 [2274–7036]
Eosinophils	204 [182–305]	164 [58–367]	184 [48–412]	171 [38–369]	135 [16–416]
Basophils	40 [12–81]	24 [<0.1–57]	44 [22–72]	35 [<0.1–88]	42 [8.2–77]
Monocytes	360 [239–388]	441 [104–1073]	382 [220–985]	288 [84–545]	**475 * [142–728]**
Dendritic cells	15 [4.9–31]	10 [3.5–43]	19 [11–50]	14 [<0.1–35]	13 [3.4–36]
Lymphocytes	1525 [1388–2412]	2052 [1196–4001]	2009 [1516–5103]	1747 [1029–3749]	2030 [418–3823]
T cells	1253 [866–1769]	1813 [879–3047]	1618 [965–3018]	**1201 ** [805–2758]**	1373 [275–2698]
TCRαβ CD4^{+}	693 [472–1082]	839 [461–1137]	868 [553–1572]	**669 ** [291–1186]**	665 [171–1483]
TCRαβ CD8^{+}	483 [244–606]	606 [211–1819]	642 [204–1558]	436 [140–1016]	448 [72–999]
CD4^{-} CD8^{-}	64 [38–73]	126 [28–214]	58 [14–190]	59 [14–150]	47 [2.9–254]
TCRαβ CD4^{-} CD8^{-}	NAv	32 [2.9–48]	21 [19–23]	25 [17–30]	13 [13–13]
TCRγδ	NAv	117 [23–206]	94 [93–96]	70 [44–161]	241 [241–241]
NK cells	343 [76–731]	190 [134–478]	317 [144–1854]	315 [109–1196]	424 [57–874]
B cells	153 [87–427]	188 [68–478]	199 [68–347]	130 [34–289]	86 [23–316]
PC	0.70 [0.32–1.9]	1.8 [0.60–6.6]	1.4 [<0.1–6.3]	1.2 [0.32–2.5]	1.1 [<0.1–3.3]

Results expressed as median [5th-95th percentile] absolute cell counts /µL of blood. Minimum and maximum values match those of the 5th and 95th percentiles, respectively. *p <0.05 and **p ≤0.01 versus the previous age group, highlighted in **bold**. Abbreviations in alphabetical order: **LST:** Lymphocyte Screening Tube; **MBL^lo:** CLL/SLL-type low-count monoclonal B-cell lymphocytosis; **n:** number; **NA:** not applicable; **Nav:** not available; **NK:** natural killer; **PC:** plasma cell; **TCR:** T-cell receptor; **y:** years.

TABLE S6. Distribution of the major populations of blood leukocytes identified with the EuroFlow LST antibody combination in MBL^{lo} subjects grouped by age and sex.

	Age group									
	40-49y (*n* = 5)		50-59y (*n* = 12)		60-69y (*n* = 17)		70-79y (n = 32)		≥80y (*n* = 18)	
Populations	M (*n* = 2)	W (*n* = 3)	M (*n* = 6)	W (*n* = 6)	M (*n* = 6)	W (*n* = 11)	M (*n* = 13)	W (*n* = 19)	M (*n* = 7)	W (*n* = 11)
Leukocytes	7605 [7360-7850]	5630 [4650-8540]	8295 [5470-13000]	6850 [4700-7990]	6410 [5570-7700]	5690 [4770-8470]	6720 [3770-8400]	5820 [3650-11630]	7080 [5990-10140]	5940 [3850-8820]
Neutrophils	5048 [4821-5274]	3626 [2502-5605]	4807 [2925-7887]	4098 [3016-5655]	3413 [2903-4147]	3012 [1681-4168]	3635 [2161-6383]	3877 [1753-7313]	4539 [2436-7036]	3241 [2274-6875]
Eosinophils	199 [182-216]	204 [184-305]	211 [82-309]	135 [58-367]	215 [79-352]	127 [48-412]	195 [52-375]	168 [20-335]	183 [90-278]	132 [16-416]
Basophils	26 [12-40]	42 [15-81]	29 [<0.1-57]	24 [12-43]	54 [22-72]	38 [23-53]	34 [<0.1-59]	40 [<0.1-111]	41 [26-66]	46 [8.2-77]
Monocytes	361 [360-363]	324 [239-388]	617 [282-1073]	339 [104-635]	417 [275-511]	307 [220-985]	324 [33-545]	283 [131-626]	**560 ** [332-728]**	426 [142-680]
Dendritic cells	21 [11-31]	15 [4.9-17]	10 [3.7-43]	12 [3.5-31]	29 [11-50]	19 [11-38]	11 [<0.1-26]	14 [<0.1-38]	16 [11-36]	11 [3.4-31]
Lymphocytes	1948 [1483-2412]	1525 [1388-2315]	2264 [1694-4001]	1601 [1196-2574]	2000 [1627-3048]	2357 [1516-5103]	1712 [1001-3749]	1774 [1029-4118]	2084 [1233-3823]	1976 [418-2739]
T cells	1060 [866-1253]	1296 [968-1769]	1892 [1224-3047]	1251 [879-1917]	1548 [1320-2428]	1902 [965-3018]	1061 [805-3189]	1246 [712-2758]	1182 [798-2698]	1631 [275-1994]
TCRαβ CD4$^+$	582 [472-693]	746 [676-1082]	900 [531-1070]	677 [461-1137]	819 [707-1572]	1040 [553-1476]	**654 ** [279-1080]**	684 [489-2045]	650 [324-1483]	906 [171-1407]
TCRαβ CD8$^+$	384 [286-483]	509 [244-606]	800 [211-1819]	451 [224-726]	664 [485-869]	588 [204-1558]	486 [140-1214]	384 [102-1016]	481 [131-999]	354 [72-689]
CD4$^-$ CD8$^-$	69 [64-73]	43 [38-71]	149 [43-209]	89 [28-214]	41 [14-190]	70 [18-115]	52 [14-88]	60 [8.2-168]	46 [10-254]	47 [2.9-243]
TCRαβ CD4$^-$ CD8$^-$	NAv	NAv	32 [2.9-48]	NAv	NAv	21 [19-23]	20 [20-20]	28 [17-30]	13 [13-13]	NAv
TCRγδ	NAv	NAv	117 [23-206]	NAv	NAv	94 [93-96]	46 [46-46]	95 [44-161]	241 [241-241]	NAv
NK cells	620 [510-731]	289 [76-343]	231 [134-478]	186 [134-243]	335 [144-1138]	317 [167-1854]	274 [109-1286]	384 [81-921]	446 [281-874]	308 [57-846]
B cells	257 [87-427]	153 [130-203]	162 [68-222]	250 [130-478]	139 [68-233]	224 [113-347]	*71 [33-251]*	*190 ## [65-300]*	84 [23-316]	87 [24-270]
PC	1.1 [0.32-1.9]	0.70 [0.37-1.0]	1.8 [0.9-2.9]	1.9 [0.60-6.6]	*2.6 [1.6-6.3]*	*0.81 ## [<0.1-1.7]*	1.6 [0.59-3.4]	0.94 [0.12-2.3]	1.4 [<0.1-3.3]	0.78 [<0.1-2.6]

Results expressed as median [5th-95th percentiles] absolute cell counts /μL of blood. Minimum and maximum values match those of the 5th and 95th percentiles, respectively. Absolute cell counts (/μL) were compared with those of the preceding age group ($^*p$ <0.05 and $^{**}p$ ≤0.01 vs. the previous age group, highlighted in **bold**) and by gender within the same age group ($^{\#}p$ <0.05 and $^{\#\#}p$ ≤0.01 male vs. female; depicted in *italics* and blue), respectively. Abbreviations in alphabetical order: **LST:** Lymphocyte Screening Tube; **M:** men; **n:** number; **MBL^{lo}:** CLL/SLL-type low-count monoclonal B-cell lymphocytosis; **NAv:** not available; **NK:** natural killer; **PC:** plasma cells; **TCR:** T-cell receptor; **W:** women; **y:** years.

TABLE S7. List of primary care physicians of the *Primary Health Care Group of Salamanca for the Study of MBL* that have participated in the recruitment of donors.

Name and surname	Primary care center
Agustín Garzón Martín	C. S. Peñaranda de Bracamonte
Amparo Herrero Sánchez	C. S. Fuentes de Oñoro
Ana María Rodríguez Medina	C. S. Alba de Tormes
Ángel Carlos González Vicente	C. S. Aldeadávila de la Ribera
Araceli Pablos Regueiro	C. S. Vitigudino
Aurora Esther Ramos Mongue	C. S. Ledesma
Begoña Sánchez Alonso	C. S. Aldeadávila de la Ribera
Begoña Sánchez Bazo	C. S. Aldeadávila de la Ribera
Bernardo Lucio García Rodríguez	C. S. La Alberca
Carmen Elena Roselló	C. S. Vitigudino
Eloy Franco Esteban	C. S. Pizarrales
Fernando Sanz Santa-Cruz	C. S. Capuchinos
Francisco Gómez Sánchez	C. S. Periurbana Norte
Francisco Macías Kuhn	C. S. Ledesma
Francisco Soto Jiménez	C. S. Santa Marta de Tormes
Horacio Marcos Vicente López	C. S. Aldeadávila de la Ribera
J. Antonio Pascual Martín	C. S. Fuentes de Oñoro
Javier Cortina Romo	C. S. Sancti Spiritus
Javier Romo Cortina	C. S. Tejares (Elena Ginel Díez)
Jesús de Pedraza García	C. S. Lumbrales
José Manuel Guarido Mateos	C. S. Vitigudino
José María Casado Romo	C. S. Alba de Tormes
Juan Montero Luengo	C. S. San Juan
Luis Cabo Sastre	C. S. Ledesma
Luis Pastor Alcalá	C. S. Vitigudino
M. Sebastián Vicente Santos	C. S. Aldeadávila de la Ribera
Manuel Pérez García	C. S. Alba de Tormes
Manuel Pérez Díaz	C. S. Pizarrales
Manuel Ramos Arranz	C. S. Ledesma
Margarita Rodríguez Vegas	C. S. Ledesma
María Auxiliadora Velasco Marcos	C. S. Tejares (Elena Ginel Díez)
María Carmen Asensio Oliva	C. S. Santa Marta de Tormes
María Concepción García García	C. S. Guijuelo
María Jesús Hernández Sánchez	C. S. Vitigudino
María Josefa Hernández Ruano	C. S. Garrido Norte (Sisinio de Castro)
María Monserrat Alonso Martín	C. S. Fuentes de Oñoro
María Paz Muriel Díaz	C. S. Miguel Armijo
María Salud Márquez Velasco	C. S. Sancti Spiritus
María Teresa Carreño Luengo	C. S. Ledesma
María Teresa Prieto Gutiérrez	C. S. Peñaranda de Bracamonte
Miguel Merino Palazuelo	C. S. Fuentes de Oñoro
Nicolás Sánchez White	C. S. Sancti Spiritus
Pablo Mateos Rubio	C. S. Ledesma
Paulino Fernández Navarro	C. S. Ledesma
Pilar Bárez Hernández	C. S. Garrido Sur
Rafael Sandín Pérez	C. S. San José (Casto Prieto)
Ricardo Herráez Martín	C. S. La Alberca
Rosalía Gómez Cabrera	C. S. Garrido Sur
Rosario Goenaga Andrés	C. S. Ledesma
Rubén Miguel Lozano	C. S. Garrido Norte (Sisinio de Castro)
Teresa Basa Jimeno Cascón	C. S. Tejares (Elena Ginel Díez)

SUPPLEMENTARY FIGURES

FIGURE S1. Summary of the statistically significant differences identified in absolute cell counts for the major populations of blood leukocytes identified with the EuroFlow LST antibody combination in non-MBL HDs throughout adulthood.

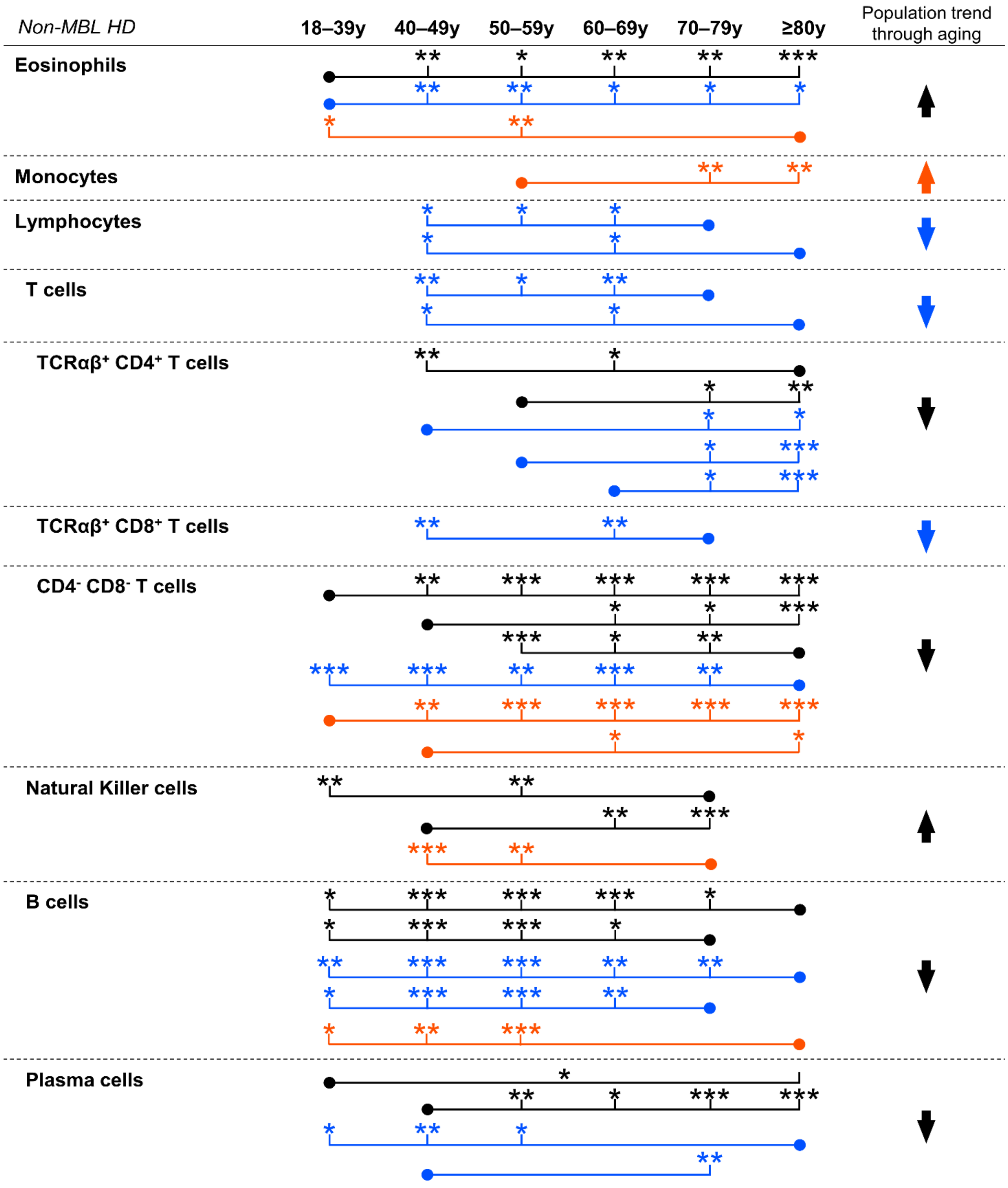

All statistically significant differences identified in the comparison of each age group against every other group are represented here. In the horizontal lines, dots represent the reference age group, whilst vertical lines correspond to those age groups that resulted statistically significantly different from the reference values. Black, blue, and orange lines correspond to the whole cohort, men and women, respectively. The right column represents the overall trend of the population cell counts through adulthood. The orange and blue arrows indicate the overall trend corresponding to the women and men groups, respectively. **p* <0.05, ***p* ≤0.01, and *** *p* ≤0.001, respectively. Abbreviations in alphabetical order: **HD:** healthy donor; **LST:** Lymphocyte Screening Tube; **MBL[lo]:** CLL/SLL-type low-count monoclonal B-cell lymphocytosis.

FIGURE S2. Summary of the statistically significant differences identified in absolute cell counts for the major populations of blood leukocytes identified with the EuroFlow LST antibody combination in MBL^{lo} subjects through adulthood.

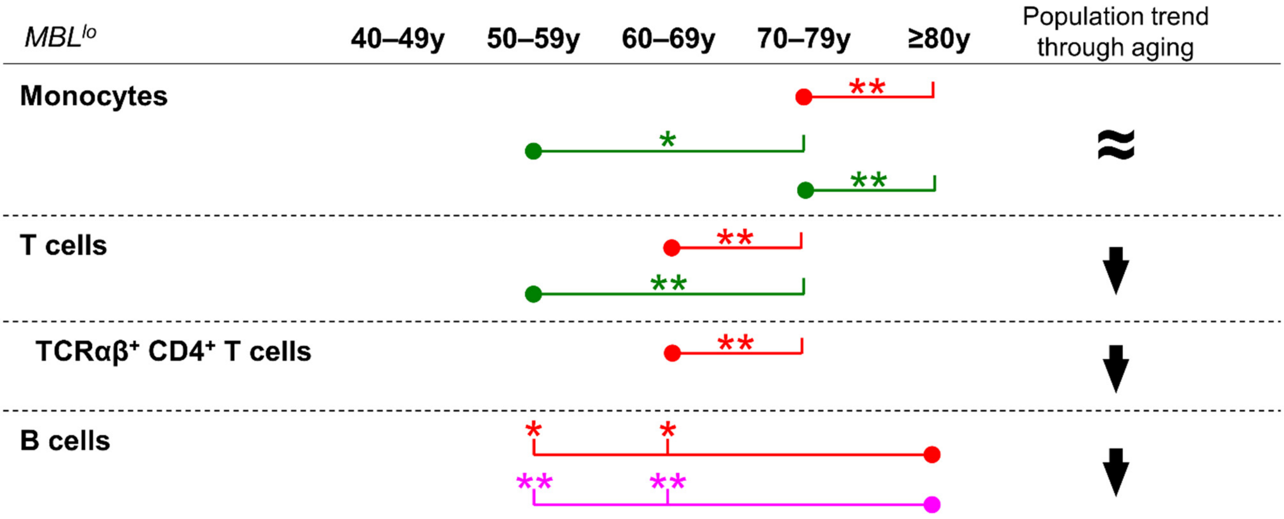

All statistically significant differences identified in the comparison of each age group against every other group are represented here. In the horizontal lines, dots represent the reference age group, whilst vertical lines correspond to those age groups that resulted statistically significantly different from the reference values. Red, green, and magenta lines correspond to the whole cohort in men and in women, respectively. The right column represents the overall trend of the population cell counts through adulthood. *p <0.05, and **p ≤0.01, and respectively. Abbreviations in alphabetical order: **LST:** Lymphocyte Screening Tube; **MBL^{lo}:** CLL/SLL-type low-count monoclonal B-cell lymphocytosis.
